# Exploring the Possibility of RNA in Diverse Biological Processes

**DOI:** 10.3390/ijms241310674

**Published:** 2023-06-26

**Authors:** Yanchen Liu, Yajing Hao

**Affiliations:** 1 Key Laboratory of Genomic and Precision Medicine, Beijing Institute of Genomics, and China National Center for Bioinformation, Chinese Academy of Sciences, Beijing 100101, China; lyccaas@163.com; 2 Department of Cellular and Molecular Medicine, Institute of Genomic Medicine, University of California San Diego, La Jolla, CA 92093, USA

The total amount of RNA in a cell is 5 to 10 times greater than that of DNA. As elucidated by the “Central Dogma” principle, RNA serves as a bridge to facilitate the important translation process of converting genetic information from DNA into protein. Apart from messenger RNAs that encode proteins, non-coding RNAs constitute 90% of RNA molecules in cells, exerting crucial regulatory roles in the modulation of gene expression and participating significantly in diverse biological processes [1,2]. Ongoing advancements in RNA research have led to the discovery of an expanding repertoire of RNA types characterized by distinct attributes, prompting further investigations into their biological functions. Currently, research in the field of RNA is predominantly focused on the elucidation of RNA biogenesis pathways, discerning the intricate structural aspects of RNA, comprehending the multifarious functions exhibited by distinct RNA types, examining the involvement of RNA in pathological conditions, and developing innovative therapeutic strategies centered on RNA-based interventions and RNA-targeted modalities. Continuously exploring the significance and functions of RNA in different biological processes not only facilitates a more profound understanding of the intricate characteristics exhibited by RNA molecules but also holds substantial implications for the advancement of varied strategies concerning the diagnosis and treatment of diseases.

The Special Issue of the International Journal of Molecular Sciences entitled “Exploring the Possibility of RNA in Diverse Biological Processes” contains original articles and two comprehensive reviews. These publications under this issue encompass various aspects of RNA biology including gene transcription [3], RNA processing [4,5], and post-transcriptional regulation [6,7], as well as the application of the state-of-art research strategies and technologies involved in RNA studies [8].

## 1. Gene Transcription

In women with preeclampsia, neutrophils infiltrate extensively into the maternal blood vessels, which triggers a sterile inflammatory response different from wound infection. Researchers have discovered that pregnancy neutrophils uniquely express protease-activated receptor 1 (PAR-1) [9,10]. This finding raises the possibility that the unique activation of neutrophils in pregnancy may be linked to a specific gene expression profile. To investigate this further, Walsh et al. conducted a study to explore whether the gene expression of pregnancy neutrophils differs when stimulated by a protease compared to bacterial lipopolysaccharide (LPS). Their findings indicated that exposure to protease resulted in three times more differentially expressed genes than LPS. The functional analysis further revealed that the protease treatment specifically enriched the MAPK signaling pathway, whereas LPS did not exhibit the same effect. These preliminary results strongly suggest that the activation of pregnancy neutrophils by protease yields a distinct gene expression profile when compared to LPS, which may provide an explanation for the systemic and unique nature of the sterile inflammatory response observed in preeclampsia [3].

## 2. RNA Processing

During the biogenesis of ribosomes in eukaryotes, numerous assembly factors (AFs) and ribosomal proteins come together with pre-ribosomal RNA (pre-rRNA) to form early ribosomal particles. These particles are subsequently transported from the nucleolus to the cytoplasm to form functional 80S ribosomes, which are responsible for protein synthesis [11]. Studies in yeast have unveiled the functions of Rrp5 in the ribosome assembly by binding to the pre-40S subunit. However, little is known about the function of Rrp5 and its partner Rok1 in multicellular eukaryotes. To address this knowledge gap, Chen et al. conducted a series of genetic and developmental experiments to elucidate the roles of Rok1 and Rrp5 in *Drosophila melanogaster* development. Specifically, Rok1 was found to be essential for the precise cellular localization of Rrp5 in the nucleolus. In the absence of Rok1 and Rrp5, the nucleolus underwent enlargement, resulting in significant disruptions to rRNA processing. The findings shed light on the vital functions of Rok1 and Rrp5 and provide insights into their implications for ribosome processing in multicellular eukaryotes [5].

In eukaryotes, the maturation of mRNA usually requires the addition of poly (A) tails to its 3′-end. A notable phenomenon observed in most eukaryotic genes is the presence of multiple potential poly (A) sites, leading to the generation of diverse transcript isoforms [12]. This phenomenon is referred to as alternative polyadenylation (APA). APA exerts regulatory control of genes in different cellular contexts by generating mRNA isoforms with variable 3’-end sequences. Wu et al. reviewed the recent achievements focusing on the molecular mechanisms underlying APA-mediated regulation of gene expression in plant stress responses. Even though further investigations are needed to unravel the detailed molecular mechanisms involved, the authors propose that APA probably serves as a positive post-transcriptional regulator in plant stress responses. Importantly, the authors highlighted the need for comprehensive studies on the mechanisms and functional regulation of APA in plants. Such studies would provide valuable insights into the regulation of plant responses to adversity and contribute to the sustainable development of agriculture [4].

## 3. Post-Transcriptional Regulation

RNA helicases are a vibrant group of RNA-binding proteins (RBPs) present in eukaryotes. They play crucial roles in various aspects of RNA metabolism, including RNA splicing, RNA export, and RNA turnover [13]. Among these helicases, DDX6 belongs to the prominent family of RNA helicases known as DEAD-box proteins. These proteins have been extensively studied across different species due to their conserved functions in the cytoplasm [14]. To unravel the role of DDX6 within the nuclei, Shih et al. systematically identified the DDX6 interactors in a HeLa nuclear extract by combining the mass spectrometry (MS) analysis with anti-DDX6 immunoprecipitation. The study revealed that two RNA-editing enzymes, namely adenosine deaminases acting on RNA (ADAR), are associated with DDX6. Furthermore, the study demonstrated the involvement of DDX6 and ADARs in retinoic acid (RA)-induced neuronal differentiation of human SH-SY5Y cells. In summary, the findings unveiled the impact of DDX6 on the regulation of cellular ADAR1/2-mediated RNA editing levels and its contribution to neuronal differentiation. These discoveries have advanced our understanding of the functional implications of DDX6 in RNA processing and metabolism, shedding light on its novel roles within the nuclei of human cells [6].

The scabies mite (*Sarcoptes scabiei*), an arthropod that infests the human epidermis, is responsible for skin diseases. While a few studies have examined certain RNAs and proteins of *S. scabiei*, no investigations have thus far compared the transcriptome characteristics across distinct developmental stages of this species or explored the associated regulatory molecular processes. Korhonen et al. conducted an initial transcriptomic sequencing of early and late-stage embryonic eggs, as well as adult females, of *S. scabiei*. Their investigation revealed a strong negative correlation between miRNAs and genes exhibiting decreased mRNA transcription, while a positive correlation was observed between miRNAs and genes displaying increased mRNA transcription during the developmental stages. These findings provide evidence for post-transcriptional regulation mediated by miRNAs during *S. scabiei* development. They also list six key sets of miRNAs, proposed to be essential regulators of differentiation and development, with potential roles in stress responses and environmental adaptation. These analyses can guide forthcoming laboratory examinations pertaining to the regulation of miRNAs (microRNAs) across all developmental phases of the scabies mite [7].

## 4. New Technologies in RNA Studies

Single-cell RNA sequencing (scRNA-Seq) has emerged as a prominent sequencing technology in recent years, offering new possibilities for investigating various facets of life science. By facilitating high-throughput and multidimensional analysis of individual cells, scRNA-Seq enables more accurate classification of cell subpopulations and in-depth exploration of cellular heterogeneity. This technology has proven invaluable in unraveling the complexity of biological systems and has found widespread application in diverse areas of research. In this review, Wang et al. provided a detailed account of the developmental trajectory and specific workflow of single-cell sequencing. The authors meticulously elucidate the intricate steps involved in this cutting-edge technology. Furthermore, they emphasize the wide-ranging applications of scRNA-Seq in recent research, with particular emphasis on its utility in investigating the tumor microenvironment. In addition, the article specifically highlights the pioneering applications and potential advancements of scRNA-Seq in the realm of traditional Chinese medicine (TCM) research. It delves into areas such as TCM syndrome differentiation studies, uncovering the mechanisms of action and efficacy of TCM, and elucidating the toxicological mechanisms associated with TCM usage. The authors also addressed crucial considerations for refining scRNA-Seq methodologies and techniques to effectively address existing challenges [8].

## 5. Discussion

The articles published within this Special Issue provide remarkable insights into diverse facets of RNA research. However, it is important to acknowledge that there exist numerous intriguing and noteworthy aspects of RNA research that have not been covered within our special issue. Currently, we would like to highlight several areas within the field of RNA research that merit attention and further exploration.

Firstly, the advent of high-throughput sequencing technologies has led to the identification of a plethora of regulatory RNAs, such as HARRs [15] and circular RNAs [16]. This expanding repertoire of non-coding RNAs underscores the remarkable diversity and complexity of RNA molecules within cells. However, there is an urgent need to unravel the functions and regulatory mechanisms associated with these non-coding RNAs. Their roles in cellular processes and their potential contributions to various biological phenomena necessitate further investigation and elucidation.

Secondly, noteworthy advancements have been made in the development of novel technologies that hold immense potential for advancing our understanding of RNA biology and unveiling new principles of RNA regulation. These include third-generation sequencing technologies [17,18], spatiotemporal single-cell transcriptomics [19], and living-cell imaging [20]. Leveraging these cutting-edge technologies offers unprecedented opportunities to revisit fundamental questions in RNA research that warrant further exploration and align with the focus of our special issue. By harnessing the capabilities of these state-of-the-art tools, we can delve deeper into the intricacies of RNA-related inquiries, uncovering novel insights and expanding the frontiers of knowledge in this dynamic field.

Thirdly, the remarkable achievement of developing mRNA vaccines in response to the COVID-19 pandemic has reinvigorated interest in RNA therapeutics [21]. Moreover, RNA-based therapeutic interventions have demonstrated remarkable success in treating rare diseases, particularly those affecting the neurological and hepatic systems [22,23,24]. In comparison to traditional protein-targeted and DNA-based drugs, RNA-based therapeutics offer numerous advantages attributed to their distinctive physicochemical and physiological properties. These advantages include the ability to target previously deemed undeliverable targets, minimal genotoxic effects, superior long-term efficacy, and the potential to address a broader spectrum of rare diseases [25,26]. At present, an array of novel RNA-based drugs has been approved [27,28], propelling RNA to the forefront of drug research. With the rapid advancement of RNA therapeutics, we anticipate increased diversification in RNA-based drugs and the development of more sophisticated drug delivery methods. 
